# Supplementary material for: Seminoma and Embryonal Carcinoma Footprints Identified by Analysis of Integrated Genome-Wide Epigenetic and Expression Profiles of Germ Cell Cancer Cell Lines
Source: PLoS One. 2014 Jun 2;9(6):e98330. doi: 10.1371/journal.pone.0098330 (PMC4041891; doi:10.1371/journal.pone.0098330)
Supplement: File S1 — ZIP file containing DMRforPairs output for significant regions. Please start from the html files. (ZIP) [file pone.0098330.s008.zip › figures/18455.pdf]

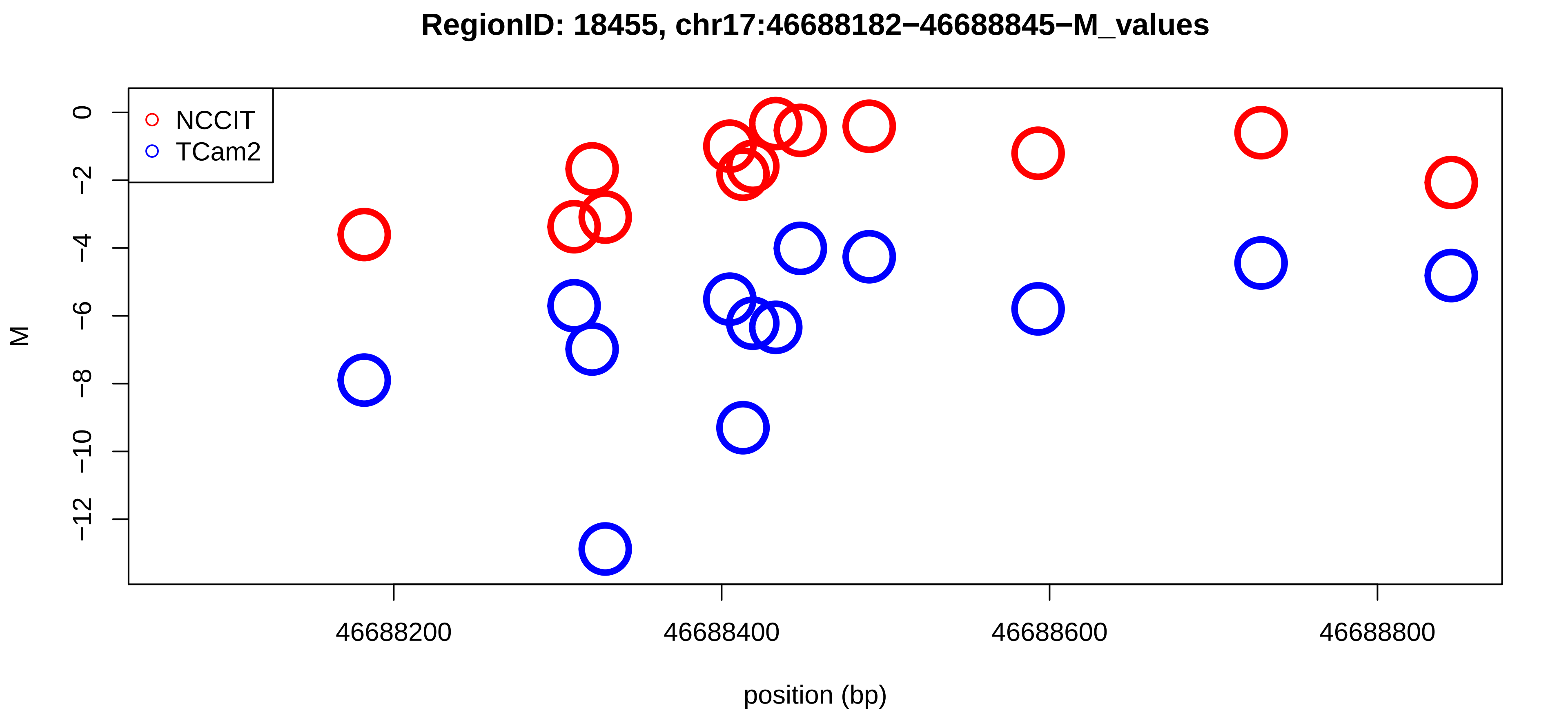
RegionID: 18455, chr17:46688182−46688845−M_values
NCCIT
TCam2
M
0
−2
−4
−6
−8
−10
−12
46688200
46688400
46688600
46688800
position (bp)

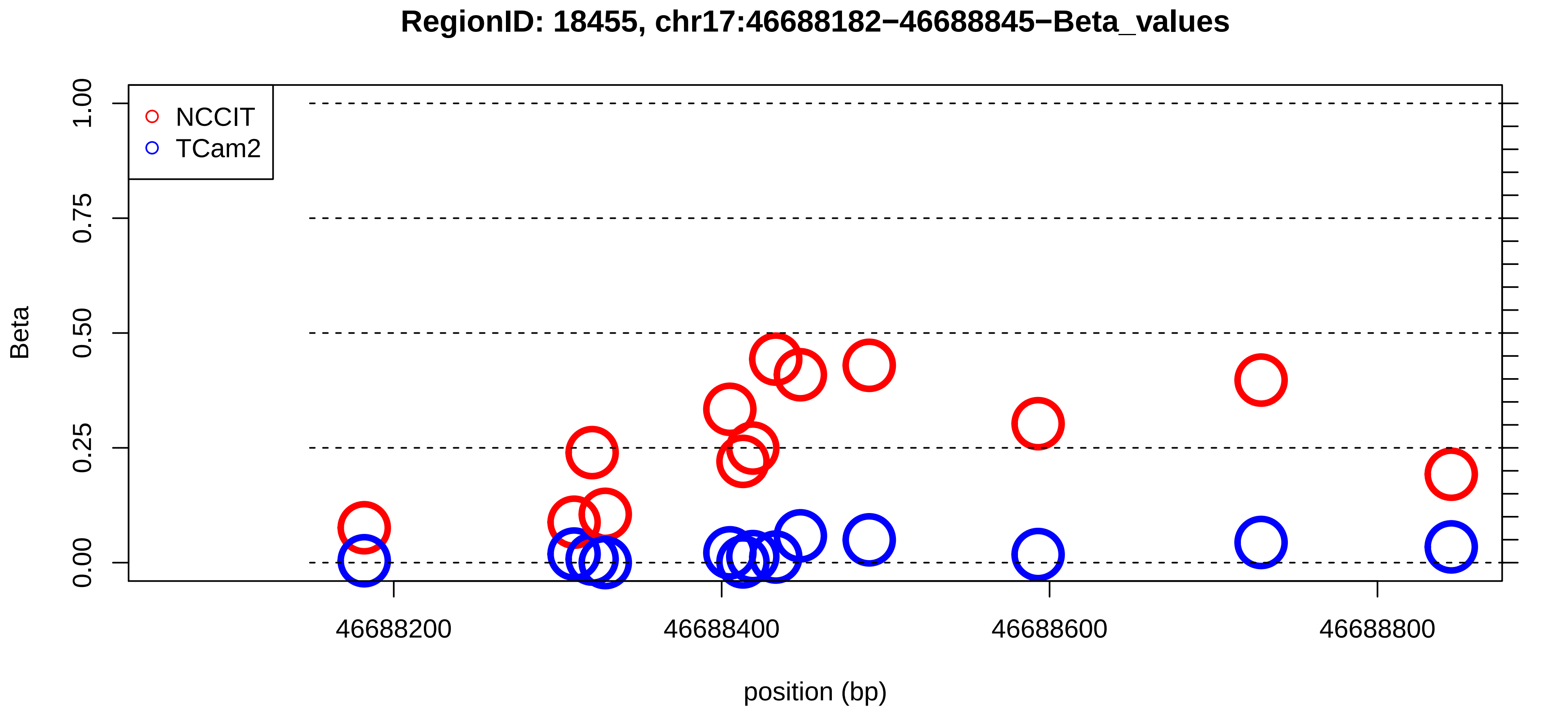
RegionID: 18455, chr17:46688182−46688845−Beta_values
NCCIT
TCam2
Beta
1.00
0.75
0.50
0.25
0.00
46688200
46688400
46688600
46688800
position (bp)

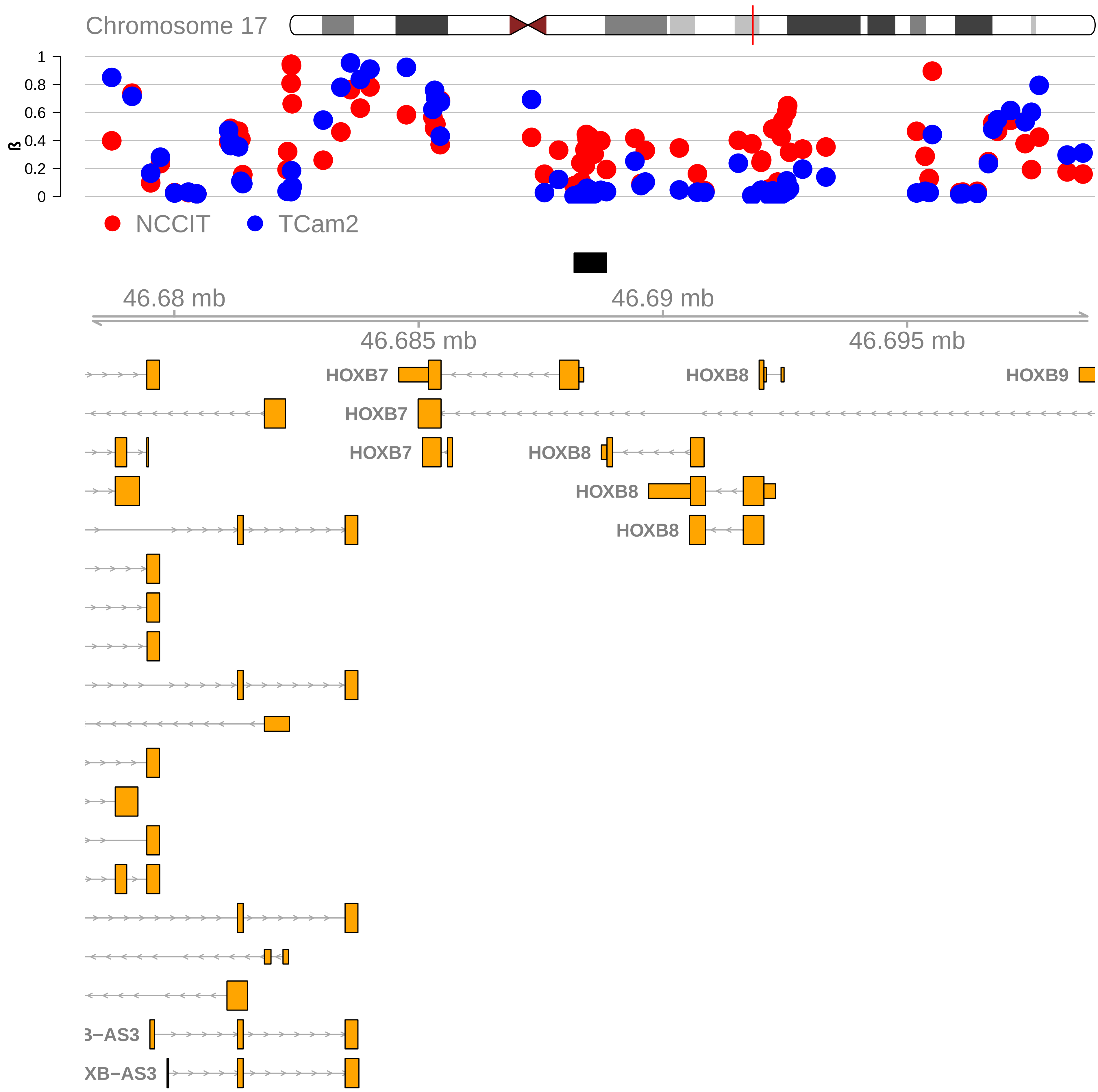

Chromosome 17
1
0.8
0.6
0.4
0.2
0
ß
NCCIT
TCam2
46.68 mb
46.69 mb
46.685 mb
46.695 mb
HOXB7
HOXB8
HOXB9
HOXB7
HOXB7
HOXB8
HOXB8
HOXB8
B−AS3
XB−AS3
